# Supplementary material for: Mollification of Doxorubicin (DOX)-Mediated Cardiotoxicity Using Conjugated Chitosan Nanoparticles with Supplementation of Propionic Acid
Source: Nanomaterials (Basel). 2022 Jan 31;12(3):502. doi: 10.3390/nano12030502 (PMC8838624; doi:10.3390/nano12030502)
Supplement: Supplementary file 1 [file nanomaterials-12-00502-s001.zip › nanomaterials-1503091-SI.pdf]

nanomaterials

Supplementary Materials

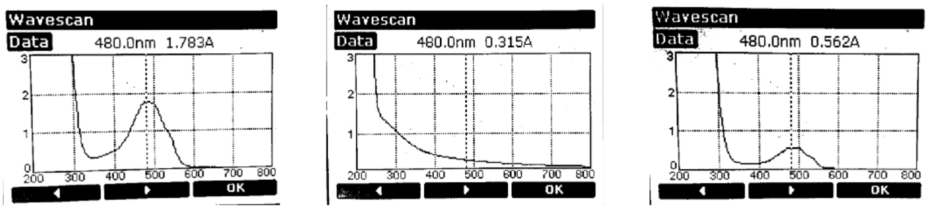

DOX   CNPs   DCNPs

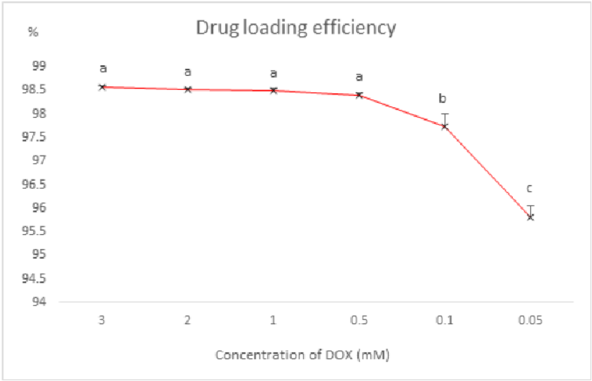

Figure S1. λmax wavelength of DOX, CNPs and DCNPs and Drug loading efficiency of CNPs at varying concentration of DOX.

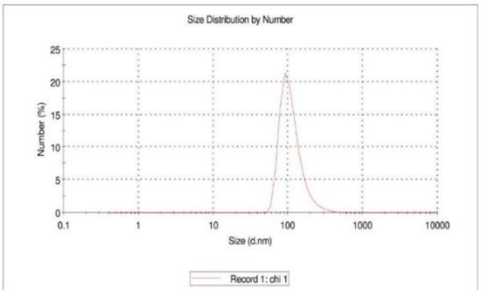

CNPs

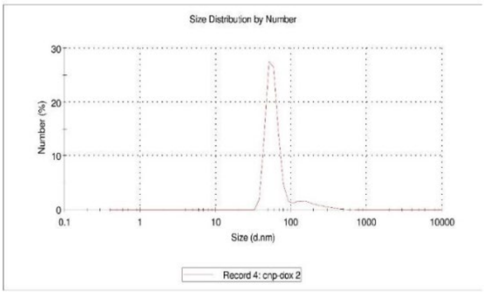

DCNPs

Figure S2. Size Distribution of CNPs and DCNPs by Number using Dynamic Light Scattering Analysis.

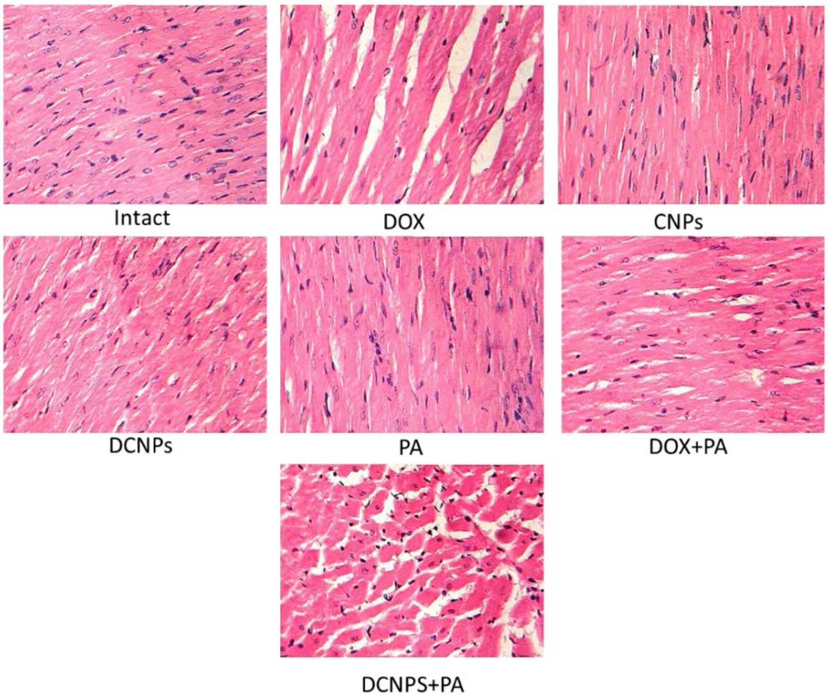

Figure S3. Histological view of Heart from experimental animals viewed under the light microscope at 40X magnification.

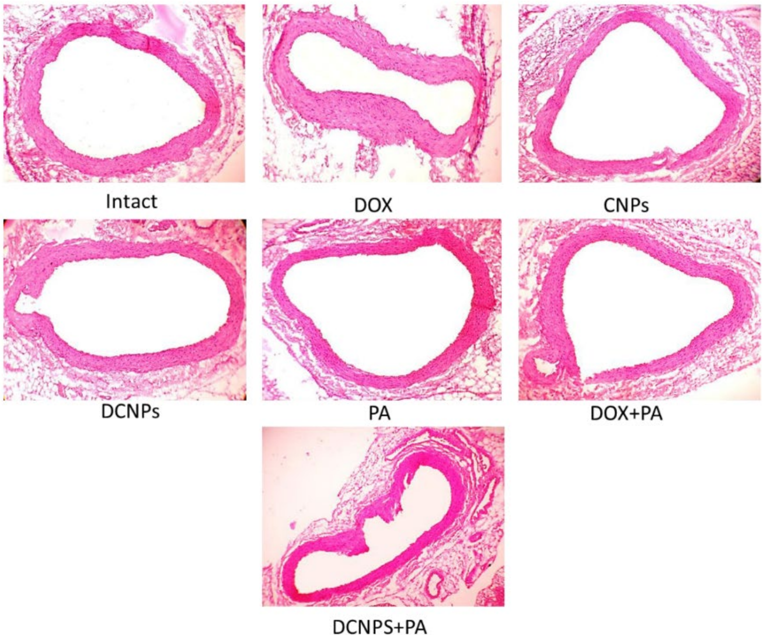

Figure S4. Histological view of Aorta from experimental animals viewed under the light microscope at 10X magnification.
